# Nurturing humanism and professionalism in a clinical setting: A multicenter study to develop a framework for a learning module for clinical students

**DOI:** 10.1371/journal.pone.0313525

**Published:** 2024-11-22

**Authors:** Rita Mustika, Anyta Pinasthika, Nadia Greviana, Eti Poncorini Pamungkasari, Annang Giri Moelyo, Rahma Tsania Zhuhra

**Affiliations:** 1 Medical Education Collaboration Cluster, Indonesian Medical Education and Research Institute (IMERI), Faculty of Medicine Universitas Indonesia, Jakarta, Indonesia; 2 Department of Public Health, Faculty of Medicine Universitas Sebelas Maret, Solo, Indonesia; 3 Department of Pediatrics, Faculty of Medicine Universitas Sebelas Maret, Solo, Indonesia; 4 Department of Medical Education, Universitas Andalas, Padang, Indonesia; Ankara University Faculty of Medicine: Ankara Universitesi Tip Fakultesi, TÜRKIYE

## Abstract

**Introduction:**

Professionalism represents a contract between physicians and society, with humanism at its core. Humanism must be developed in medical education, especially in clinical settings, as students meet real-life professional situations. However, dynamic and unpredictable clinical settings might expose students to authentic yet unexpected far-from-ideal situations that might hinder the humanism and professionalism process. Furthermore, culture plays an influential role, highlighting the importance of developing an appropriate and contextual learning strategy. Hence, this study aims to conceptualize strategies to teach humanism and professionalism in high-power distance and collectivistic settings.

**Methods:**

This multicenter qualitative phenomenological study used maximum variation sampling to recruit participants, consisting of clinical students, teachers, and coordinators from three medical schools in Indonesia. Data were collected through focus group discussions (FGDs) and in-depth interviews. Thematic analysis was conducted using the steps of coding and theorization methods.

**Results:**

A total of 15 FGDs and one in-depth interview were conducted with 57 students, 39 teachers, and 18 coordinators. Socialization theory was used to analyze themes, leading to a culturally related approach to learning. Professional identity formation (PIF) was highlighted as the center of learning humanism and professionalism, with character building as the main principle. Designing a curriculum for humanism and professionalism for clinical learning should consider the longitudinal nature of PIF and ensure that professionalism explicitly exists in the curricula. As cultural impact might pose challenges, it must also be acknowledged and addressed. Meaningful integrated learning experiences, patient exposure, and reflection serve as the cornerstones of teaching–learning strategies while considering longitudinally explicit assessments.

**Conclusion:**

Nurturing humanism and professionalism in undergraduate clinical settings is a longitudinal character-building process, with PIF as its center. Longitudinal, explicit, and dynamic strategies should be considered as part of the framework of teaching–learning and assessment of humanism and professionalism, as well as faculty development efforts with close attention to cultural factors.

## Introduction

Humanism and professionalism are essential aspects of medical practice. Professionalism is the foundation of medical doctors’ social contract with society. The American Board of Family Medicine defines medical professionalism as a belief system that group members declare to one another and the public, the shared competency standards and ethical values they promise to uphold in their work, and what the public and patients should expect from medical professionals [1]. Humanism is defined as intrinsic values that manifest as the core of medical professionalism. These values include integrity, compassion, altruism, respect, and empathy. It is believed that a humanistic person can become a genuine professional doctor [2].

Humanism improves clinicians’ satisfaction and reduces practical mistakes [3]. Without it, medical practice would be inadequate [4]. Unfortunately, when new technologies and medical breakthroughs emerge, they are typically followed by increased burnout rates and decreased empathy among medical students. Furthermore, humanism in medical care appears to be declining [5, 6]. The degradation of humanism in medical care in recent years has raised interest in teaching humanism to medical students [7].

The clinical setting is the most suitable learning environment to instill humanism and professionalism in medical students. Medical students enter clinical settings during the professional phase of their education, right after the academic phase. In clinical settings, medical students can directly engage in real-life situations to learn about the physician–patient relationships from the clinical teacher, who serves as the role model, and reflect on their experiences accordingly [7, 8]. A previous study demonstrated that learning professionalism could be implemented in both formal and informal curricula [9]. Role model exposure provides medical students with an opportunity to explore and reflect on professional values and determine which values they should embody as professional doctors [10]. However, when serving as role models for humanism, clinical teachers do not always function under ideal conditions; one study highlighted a gap between ideal and actual situations [11]. Therefore, institutional support and faculty development are other essential aspects of teaching professionalism that must be considered [12].

Although students are exposed to actual health service situations in clinical settings, factors such as the clinical work rhythm, exposure to negative role models, and students’ personal backgrounds can become obstacles to nurturing humanism [8]. The nurturing of humanism and professionalism are highly influenced by learning about cultures across contexts. For instance, in the learning context in high-power and collectivistic culture settings with a hierarchical student–teacher relationship, students had difficulties asking for individual feedback and seeking clarification on ethical dilemmas they faced in clinical settings [13]. Therefore, to overcome obstacles in professional learning, an appropriate learning strategy is needed [14].

As nurturing humanism and professionalism is integrated into the professional identity formation (PIF) process, the socialization process of medical students in the learning environment is emphasized. Upon entering medical school, medical students, who have preexisting personal identities, acquire professional identities not only through learning medical knowledge but also by socializing with teachers, peers, and patients in the learning environment [15]. Thus, the learning strategy of fostering humanism and professionalism closely relates to local situations and cultures. According to Hofstede’s cultural dimensions, Indonesia has a high power distance index, which is characterized by an accepted inequality of power within organizations and an emotional distance between parties with high and low power. This cultural dimension impacts how teachers and students interact within the learning environment; students may feel discouraged to speak up and are expected to show moderation because of the superiority of their teachers and the existence of the subordinate nature valued in the organization. Furthermore, Indonesia has a highly collectivistic culture that prioritizes connectedness and compliance with societal norms [16, 17]. Research on the topic of nurturing humanism and professionalism in the high power distance and collectivistic context is scarce. Hence, this study aimed to conceptualize how to nurture humanism and professionalism in the clinical setting in a high power distance and collectivistic cultural context. This study will provide the teaching–learning framework for nurturing humanism and professionalism in the clinical setting and explores the extent to which learning contexts and cultural aspects contribute to its process.

## Methods

### Context

This was a multicenter qualitative study involving three medical schools in three different areas in Indonesia: Universitas Andalas in West Sumatera, Universitas Sebelas Maret in Central Java, and Universitas Indonesia in Jakarta. The undergraduate program in medicine in Indonesia receives high school graduates and includes 3.5–4 years of a preclinical program followed by 1.5–2 years of a clinical program. The teaching and learning processes of humanism and professionalism in the clinical setting at the three universities are embedded in the curricula; however, the formal and explicit teaching and learning approach on this aspect must be developed. All three institutions agreed to conduct the research collaboratively to conceptualize the appropriate teaching–learning method for cultivating humanism and professionalism in clinical settings.

### Data collection

The research team consisted of five medical educationalists and two clinical teachers from three medical institutions in three areas of Indonesia. All researchers were involved in the data collection process by cross-facilitating focus group discussions (FGDs), in which a researcher from Institution A facilitated the FGD for participants at Institution B, and so forth. A briefing session was held with all researchers to align perceptions and discuss the focus group discussion guide before the sessions took place. None of the researchers acted as a research subject in this study.

A phenomenological approach was used in this study to explore the underlying phenomenon based on respondents’ experiences. In particular, data were gathered by conducting FGDs and qualitative literature reviews. The targeted study population at each medical faculty consisted of clinical students, clinical teachers, and module coordinators. A total of 15 FGDs and one in-depth interview were conducted from August to September 2022: seven FGDs with clinical students, five FGDs with clinical teachers, three FGDs with program coordinators, and one interview with the education manager. Each FGD sessions consists of 8–10 members. This study used a purposive sampling method with a maximum variation approach to select participants for each FGD until data saturation was reached. Participants were selected according to the variations considered and then invitations were sent. Participants joined the research voluntarily if they wish to accept the invitation. Variations considered for the clinical teachers were their career level, gender, and assignments in various clinical departments. Variations considered for clinical students were their gender and learning levels at each institution, while program coordinators were selected on the basis of the different clinical rotations and programs at each institution. This study acquired ethical approval from the Faculty of Medicine Universitas Indonesia Research Committee under number 22-06-0606. Written informed consent was collected from participants before data collection started. The FGDs and interviews were held online via Zoom meetings and moderated by the research teams. All moderators used guided questions for the FGDs, which can be viewed in Table 1, and the probing points about cultural aspects were embedded in the group discussion guidelines.

**Table 1:** Focus group discussion guided questions.

Questions
Opening Question
What do you think about nurturing humanism and professionalism in clinical learning?
Main Question
1. How did learning humanism and professionalism take place in your setting/institution?
Probing: How is it planned in the curriculum? What kind of learning activities/methods are implemented? How is it assessed?
2. In order to develop humanistic and professional doctors, what should be done in clinical settings?
Probing: How should it be planned in the curriculum? What is the ideal learning activity/method? What kind of assessment should be implemented?
3. What needs to be done to support the optimal learning of humanism and professionalism in clinical settings?
Probing: Learning resources, faculty development, facilities and funding, regulations, etc.
4. What are the possible barriers to nurturing humanism and professionalism in clinical settings?
Probing: Hidden curriculum, learning environments, student–teacher relationship

### Data analysis

The collected data were recorded, transcribed, and analyzed using the Steps of Coding and Theorization (SCAT) method to obtain categories, subthemes, and themes [18]. The data analysis was conducted iteratively. Transcripts were distributed to all authors, and the results were discussed to determine the theme and subthemes. This iterative discussion process was done four times until final themes and subthemes were achieved, and any disagreements were discussed and solved by all authors. Quotes in the results were coded according to the data source (MS: medical student, CT: clinical teacher, and PC: program coordinator) and FGD number.

## Results

**Fig 1:**
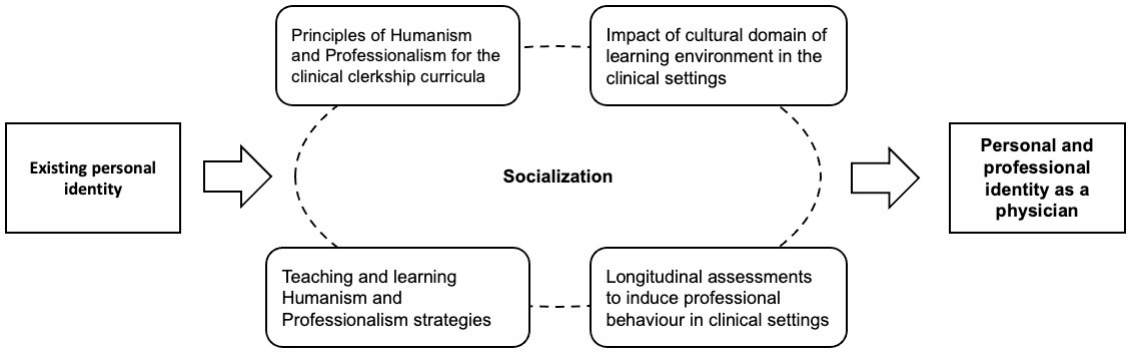
Teaching and learning framework for nurturing humanism and professionalism in clinical settings.

A total of 15 FGDs and one in-depth interview were conducted with participants from three universities in Indonesia. The group consisted of 57 medical students, 39 clinical teachers, and 18 program coordinators. The data were analyzed using the framework of socialization theory [15, 19]. The framework of the themes is presented in Fig 1.

The emergent themes were classified into the four requirements of the nurturing professionalism process proposed by Ong et al. [20] to design explicit, culturally appropriate training in humanism and professionalism that considers PIF and specific clinical learning environments. The list of themes and subthemes is provided in Table 2.

**Table 2:** Emergent themes and subthemes.

Themes	Subthemes
Professional identity formation as the center of learning humanism and professionalism	Students’ backgrounds and personal beliefs in the PIF process
	Students’ personal aspects (i.e., motivation, resilience, well-being, ability to adapt to change) in the PIF process
	Socialization process as the center of PIF
	Attributes and key features of humanistic and professional doctors as the end goal of PIF:
	(1) Adequate clinical skills
	(2) Humanistic qualities
	(3) Maintaining competence and lifelong learning
Principles of humanism and professionalism for the clinical clerkship curricula	Nurturing humanism and professionalism as a character-building process
	Societal demands of professional doctors as the background of the learning process
	• Acting professionally in the era of social media and rapid development of technology
	Nurturing humanism and professionalism as a longitudinal process
Impact of the cultural domain of learning environments in clinical settings	The need for professional behavior guidelines for stakeholders in the learning environment
	Culture of compromising unprofessional behaviors
	Generational gaps and hierarchical culture in clinical settings
	Limited opportunities for interprofessional collaborative practice in the learning environment
	Nurturing humanism and professionalism as “the second-layer teaching agenda”
Teaching and learning humanism and professionalism strategies	Meaningful integrated learning experiences with direct patient interaction
	Immersive intra- or extracurricular community activities
	Reflection and debriefing sessions of learning experiences to internalize humanism and professionalism
Longitudinal assessments to induce professional behaviors in clinical settings	Demerits of assessing professionalism as an embedded component in other assessments
	(1) Implicit nature of assessment of professionalism
	(2) The issue of fake professionalism
	(3) Professionalism is not prioritized in assessments
	Longitudinal assessment approach
	(1) The importance of hurdle assessment
	(2) Applying portfolios as learning and assessment instruments

### 1. Professional identity formation as the center of learning humanism and professionalism

PIF is the center of humanism and professionalism development in clinical students. The participants were aware that students’ PIF is an important component of teaching humanism and professionalism. The teachers highlighted the dynamic process of PIF and emphasized that each student had individual intrinsic values that they brought from their family backgrounds and personal beliefs.

*“We think that we accept students who are…‘half-done’. It means that the students already have their own basic characters and backgrounds. [They differ in] how they are raised in their family and went through [their] education.”*

*(PC, IN1)*

*“Students also have their own opinions and standards, including beliefs and religions. Now that could pose a barrier in interacting with other people. For example, when I interact with patients of the opposite sex, I feel reluctant, especially when I have to examine the patient. Maybe it’s something worth considering that some students might feel that way.”*

*(MS*, *FGD7)*

The respondents highlighted several personal aspects that affected students’ PIF, including motivation, resilience, well-being, and the ability to adapt to changes in their transition to the clinical stage.

*“How we, as health professionals, can regulate their stress and emotions so that we can communicate with patients better…even though we are in a stressful and high-pressure environment. I think it’s important.”*

*(MS, FGD12)*

*“I think the students are still…far from being a professional…because they still have to adapt [to the clinical phase]. They used to only listen to lectures, and now they have to go to the hospital; it takes time to adjust themselves. Usually, in their first year [of clinical rotations], they’re a bit puzzled and nervous when meeting patients and clinical teachers.”*

*(CT*, *FGD9)*

The students also learned about humanism and professionalism through socialization with role models and mentors as well as the direct supervision of the residents and clinical teachers around them.

*“Professionalism is not taught with words but in the form of following examples [role models], in interacting with patients, interacting and working together with other health professions to treat the patient. So, we learn by seeing and following them.”*

*(MS, FGD8)*

*“It is our chance and obligation to guide students in becoming good, honest, well-behaved doctors. The role of clinical teachers as role models is very substantial in building students’ characters.”*

*(CT*, *FGD9)*

The respondents also highlighted several key features and attributes of humanistic and professional doctors as goals of PIF. These attributes were (1) adequate clinical skills, including the ability to conduct evidence-based practice, follow medical ethics, provide patient-centered care, communicate effectively, and develop therapeutic relationships with patients and their families; (2) humanistic qualities, such as the ability to empathize with patients, manage one’s emotions, and respond to patients’ needs; and (3) the ability to maintain competence and pursue lifelong learning.

*“Professional doctors are able to apply their knowledge in medicine comprehensively to the patient.”*

*(PC, FGD11)*

*“Because we are treating patients, we need to have humanistic values. We need to treat and value patients as humans. Therefore, we need to put ourselves in their shoes and treat them just like how we want ourselves to be treated.”*

*(MS, FGD7)*

*“[Being a] professional doctor also means that they are aware of what they are lacking and are willing to improve themselves in the future. What matters is the mindset that we can still improve ourselves, and there are a lot of things we need to learn.”*

*(MS*, *FGD3)*

### 2. Principles of humanism and professionalism for the clinical clerkship curricula

The participants indicated that humanism and professionalism are core competencies that must be included in the curriculum. They viewed the cultivation of humanism and professionalism as continuation of students’ character building so that they become medical doctors who value patients as human beings.

*“A very important note: what students really need in nurturing professionalism is character. Building students’ characters is the main point.”*

*(CT*, *FGD4)*

The medical schools participating in this study demonstrated their attempts to incorporate humanism and professionalism longitudinally across their curricula. They also highlighted the societal demands of professional doctors as one of the main reasons why this domain must be nurtured.

*“The public demands the kinds of qualities [possessed] by doctors in Indonesia that are deemed professional. I think that becomes a factor that motivates us to maintain professionalism. At least we’d know what kind of doctor the public wants. So, we have to improve ourselves [in terms of professionalism] and improve our education on that aspect.”*

*(CT*, *FGD6)*

However, the participants also highlighted the current rapid development of technology, especially following the emergence of the COVID-19 pandemic. This has brought about the need for new skill sets related to professionalism, which must be cultivated in medical schools, especially regarding the ability to act accordingly and professionally in the era of social media and healthcare-related technologies (e.g., telemedicine).

*“Nowadays, we can find everything happening on social media, so we need to be careful when practicing as a medical doctor; we need to be professionals.”*

*(MS, FGD13)*

*“Technology is growing fast, and there are lots of medical technologies—[smartphone] applications, telemedicine; maybe one of the qualities that current doctors need is humanistic values. Therefore, what makes us different as doctors, whether through face-to-face or through telemedicine applications, are those humanistic values.”*

*(MS*, *FGD7)*

The respondents highlighted the attempts of medical schools to longitudinally nurture humanism and professionalism through explicit teaching in the preclinical stage. Students were also aware that humanism and professionalism should be learned and developed during their studies, not only in the preclinical stage but also in the clinical stage. Furthermore, they agreed that humanism and professionalism should be explicitly taught in the clinical stage.

*“During the preclinical EEP [empathy, ethics, and professionalism] module, we shadowed and interviewed doctors about ethics and stuff, and we really got the ‘show, don’t tell’. The EEP module in the preclinical phase really showed us [professionalism].”*

*(MS, FGD1)*

*“I agree that it [teaching professionalism] should be conducted longitudinally. It’ll be more effective if we can insert it into each rotation, like internal medicine and child health, so that it [professionalism] will be more specific and applicable.”*

*(MS*, *FGD2)*

### 3. Impact of the cultural domain of learning environments in clinical settings

The respondents mentioned a lack of professional behavior guidelines that must be acknowledged and followed by all stakeholders within the institutions. Such guidelines could help students and teachers behave and assess their actions accordingly, thus supporting their professional development over time. Although professionalism is listed as one of the core competencies in the national competency standard of doctors, teachers and coordinators reported difficulties in translating this into clinical rotations. The lack of such guidelines also affected how the students and teachers interacted.

*“It [professionalism] is not easy. Because we’re assessing attitudes and behaviors, it’s not like we’re assessing written exams. Maybe we need some sort of guideline for which kinds of behavior are still acceptable and can be corrected and which kinds of behavior cannot be accepted and are a sign that this student can’t become a medical doctor.”*

*(CT, FGD6)*

*“When we designed SKDI [Standar Kompetensi Dokter Indonesia—National Standards of Competencies of Medical Doctors Indonesia], professionalism was number one because we realized being professional is a requirement. But how we formulate it in the curriculum…it’s another problem.”*

*(CT*, *FGD4)*

The participants emphasized the culture of compromise as a response to unprofessional behaviors, especially regarding ethical dilemmas and negative role modeling. This is one of the challenges of nurturing humanism and professionalism in clinical settings and could further affect students’ professional development.

*“There’s a tendency to make unprofessional behavior somewhat acceptable… a lot of people [in the clinical environment] think it’s okay, and it makes us [students] think that kind of [unprofessional] behavior is okay too, even though in the beginning we know it’s unacceptable.”*

*(MS*, *FGD2)*

Participants highlighted that learning professionalism took place in a rigidly hierarchical healthcare system, owing to the generation gaps between students, residents, and faculty members. This affected the daily interactions among stakeholders in clinical settings. Furthermore, students also mentioned that the limited opportunities to be involved in interprofessional collaborative practice presented a challenge in developing their professional identities.

*“From what I see… they [students] are pressured. Clinical teachers pressured residents; residents pressured medical students. It’s like consecutive pressures.”*

*(CT, FGD14)*

*“We meet a lot of people in the hospital—not only doctors, but also nursing, midwifery, or physiotherapy students. But, in reality, we don’t have any sessions or activities that involve interacting with them. Real nurses, on the other hand, are, in a way, our teachers too. We can’t really collaborate with them because they’re our teachers.”*

*(MS*, *FGD8)*

The undergraduate medical curriculum, which tends to focus more on clinical skill attainment and clinical reasoning, has also become a challenge in fostering humanism and professionalism in clinical settings. The participants viewed nurturing humanism and professionalism as “the second-layer teaching agenda” that was not explicitly written in the curriculum; thus, these attributes were not prioritized by teachers and students.

*“Yes, we are more focused on the clinical aspect of the case. About the professionalism aspect…sometimes we skipped it a little, sorry.”*

*(CT, FGD10)*

*“From what I see when interacting with students through bedside teaching or assessments, it’s focused on clinical reasoning, knowledge like physiology, or patient management. We did assess professionalism, but we didn’t discuss it any further.”*

*(PC*, *FGD5)*

### 4. Teaching and learning humanism and professionalism strategies

Regarding the further cultivation of humanism and professionalism in the clinical stage of a student’s learning, this study highlighted the importance of meaningful integrated learning experiences with direct patient interaction, which can help develop students’ identity formation as future medical doctors. Their experiences during the COVID-19 pandemic, which limited their opportunities for direct interaction with patients and exposure to the clinical learning environment, highlighted the importance of patient involvement.

*“Professionalism can be taught [and] integrated with the ongoing clinical rotations, where students can interact with patients, participate in bedside teachings, and be assessed with mini-CEX.”*

*(CT, FGD4)*

*“Aside from teaching about the clinical content, we should also talk about how students interact with patients—how they design patient management plans that are suitable for patients’ situations while respecting patients’ values and considering their resources.”*

*(CT, FGD6)*

*“I feel that I’m still not that professional, especially regarding cases that we haven’t met yet. Our batch didn’t have any rotations aside from the main teaching hospital [due to the pandemic], so we are really lacking in experience. Medicine requires us to not only learn about illness and clinical skills but also about the social problems of patients. That’s what makes it different from textbooks. It’s a challenge for medical students in the pandemic era.”*

*(MS*, *FGD3)*

Interacting with patients through direct patient exposure is essential for nurturing humanism and professionalism among students. They also valued immersive intra- or extracurricular community activities that they were involved in as important teaching–learning components to foster humanism and professionalism. These community services could also mitigate limited patient interactions during hospital-based learning.

*“In the community health rotation, I feel more professional to patients, to the community. We were taught to work with the community, give education to the elderly, etc. We focused on preventive measures, so even though we didn’t learn much clinical knowledge, we felt connected to the community and learned how to become good doctors among the community.”*

*(MS*, *FGD8)*

The participants highlighted the importance of venues through which they could discuss and reflect on their clinical learning experiences and role models. Reflective essays, which are often requisite learning tasks in clinical rotations, were also highlighted as tools to help students internalize humanism and professionalism. The students also suggested conducting debriefing sessions to reflect on and discuss unexpected learning experiences.

*“They [students] wrote a reflection [essay]. We designed the reflection to be confirmed and discussed with supervisors.”*

*(PC, FGD11)*

*“It’d be nice if we had dedicated sessions to talk about students’ experiences in the clinical learning environment. But the session has to be safe, so it can be a safe space for them and it’s guaranteed that what is said in that room stays in there, so they can freely talk about negative experiences. The goal is to give feedback and affirmations about role models’ behaviors—positive and negative ones.”*

*(CT*, *FGD6)*

### 5. Longitudinal assessments to induce professional behaviors in clinical settings

The current practice to assess students’ professionalism and humanism is embedded in several assessment instruments, including workplace-based assessment and the Objective Structured Clinical Examination (OSCE). Because it is a component of other assessment instruments, participants reported that fake professionalism often occurs, as this attribute is only assessed during examinations.

*“From what I know, professionalism is one of the aspects that is being assessed in clinical examinations, but I feel that in these two years [of clinical clerkship], the subject of professionalism is not really touched upon…and not consistent.”*

*(MS, FGD3)*

*“We all know about fake professionalism. In front of patients, they are doing and saying things that they don’t really mean with sincerity.”*

*(CT*, *FGD6)*

The participants mentioned other challenges of this embedded assessment of humanism and professionalism; these were related to the implicit nature of such an assessment, which resulted in students behaving pragmatically. Students focused on the other items assessed in the respective instruments rather than humanism and professionalism, which are more difficult to assess.

*“A lot of students think that they only need to pass the national competency exam. However, humanism and professionalism are not [explicitly] stated in the national competence exam…our students are very pragmatic.”*

*(PC*, *FGD11)*

Therefore, the respondents suggested that longitudinal assessments of humanism and professionalism should be conducted through a longitudinal hurdle assessment approach with an emphasis on the coaching and mentoring process, which would explicitly discuss students’ humanistic values and professional behaviors over time. They also emphasized the importance of effectively applying portfolios as learning and assessment instruments in clinical settings.

*“When it [professionalism on the national competency exam] is not there…our students still need the external ‘pressure’ to increase their motivation to learn something. When it [professionalism] is not a requirement, it’s going to be hard.”*

*(PC, FGD11)*

*“I don’t know if we can see [students’ progress] from year one to year two—if it’s getting better from the start to the end of the clinical rotation. Hopefully, clinical rotation can give color and strengthen students’ professionalism…from the beginning of clinical clerkship until the end, when they’re preparing for the national competency exam.”*

*(PC*, *FGD5)*

## Discussion

Medical schools should develop and provide approaches to teaching medical science and clinical skills while developing humanism and professionalism. The development of professionalism and humanism must occur in all courses and activities throughout students’ medical education and should be explicitly included in the curriculum as early as possible [12, 21]. Teaching professionalism must begin in the preclinical phase so that students gain the necessary cognitive base, which they will apply and internalize during their experiential learning in the clinical phase [22]. However, considering the complexity of the clinical setting and the strong influence of cultural aspects, various components of teaching in the clinical setting require further exploration. The clinical phase is crucial in helping students establish a deeper understanding of how to be a physician; each learner experiences direct exposure to the actual job, interacts with real patients, and acts like a real doctor until he or she becomes one [23]. Therefore, this study explored the framework needed to develop approaches to nurturing the humanism and professionalism values of a physician during the clinical phase of learning and the extent to which cultural aspects influence the process.

The main finding of this study is that, in principle, nurturing humanism and professionalism is a character-building process that begins in early life and develops continually. Character refers to ‘the mental and moral qualities distinctive to an individual’ [24]. Findings of this study argued that students’ professional identity formation started with this character-building process, before integrating identity as a ‘doctor’. In early life, a person’s character is built through positive examples from their caregivers and surroundings, with strong influence from the culture and beliefs of one’s family. Thus, when they enter medical school, students have existing identities formed through genetic traits, previous life experiences, and social interactions (e.g., their upbringing, culture, religion, and former educational background). Their socialization experiences in their learning environments while studying medicine result in new attributes and identities as professional doctors. Cruess et al. [15, 19] conceptualized this as the socialization process of PIF.

During socialization in medical school, the intrinsic values students have gained from their families and environments are nurtured and developed so that they can acquire the attributes of professional physicians later in their careers. Cohen [2] illustrated that this process is similar to nurturing humanistic values, which ultimately become the core of professional identity development in medical school. Humanism is believed to be the soul of true professionalism; a person raised by a humanistic family will grow the seeds of humanism, which will affect their values and behaviors when they enter medical school. Eventually, if the medical school provides a humanistic environment, they will become professional physicians. Therefore, the results of this study highlight the importance of considering students’ existing personal identities while cultivating humanism and professionalism. These results are in line with studies that have focused on shifting from teaching professionalism to supporting the PIF of medical students in nurturing professionalism [15, 20, 25]. Evidence from the same setting showed that the PIF of medical students was also affected by personal factors, such as students’ motivation, resilience, and well-being [26].

Findyartini et al. [26] discussed the process of PIF as a “journey” that is initiated by the student’s motivation. Along this journey, stress and burnout are common, if not inevitable, experiences due to the long working hours, immense cognitive load, and tension from continuous identity changes during socialization. Hence, resilience and well-being are important factors in navigating dynamic identity formation [25, 26]. Thus, it may be beneficial to support students throughout their education, including the transition phase to the clinical stage, as this may promote their professionalism in the clinical setting [15, 19, 25].

Furthermore, this study emphasizes the importance of teaching humanism and professionalism longitudinally. Our results revealed that the medical education institutions involved in this study are aware of this. However, the undergraduate medical curriculum, which tends to focus more on clinical skills attainment and clinical reasoning, poses challenges to the task of nurturing humanism and professionalism in a clinical setting. In addition, attempts to nurture humanism and professionalism in the clinical setting are still made rather implicitly through direct patient interactions and role modeling from clinical teachers and residents, processes that are also central to PIF. An immersive process in which students interact with components of the learning environment and internalize professional values and norms can shape them into the professionals they aim to be. Cruess and Cruess [15, 19, 23, 25] have argued that role models and mentors as well as clinical and nonclinical experiences are the most powerful drivers of this socialization process through conscious and unconscious pathways. Therefore, such meaningful interactions with patients, clinical teachers, and peers are crucial to ensuring clinical students’ professional development and identity formation [27]. Furthermore, the “white coat ceremony,” a ritual carried out in several medical schools before students enter the clinical stage, could also be used as a ‘refresher’ in nurturing students’ humanism and professionalism before they step into the clinical phase. The ceremony could serve as a learning opportunity for students to review ethical principles mindfully, and it serves as a reminder of the humanism and professionalism concepts they learned in the preclinical stages.

However, professional dilemmas and negative role modeling are often inevitable and still exist in experiential learning in the workplace [28]. As students start to internalize values and norms that contrast their existing personal identities during socialization, they might become confused about their role and sense of self [15, 19, 25]. Therefore, engaging clinical students in reflective dialogues, assisting them in acknowledging their existing identities, and providing opportunities to internalize their experiences through guided reflection—tasks that involve role models and mentors—are crucial in supporting students’ PIF as well as their humanism and professionalism development [19, 25, 29].

Therefore, the results of this study call for robust teaching methods to teach humanism and professionalism more formally and explicitly in clinical settings. The findings of this study also revealed a culture of compromise in responding to unprofessional behaviors, which resonates with the results of a previous study conducted in a similar setting [13]. The culture of high power distance and collectivism plays a role in this regard, although Monrouxe [30] argued that power distance in a clinical setting is almost homogenous across countries. This culture in the learning environment is further intensified by the local cultural context of Indonesia that is hierarchical and collectivist in nature [16]. Another study demonstrated that senior medical students had higher degrees of power distance, collectivism, masculinity, and long-term orientation [31]. Aligning with the nature of a society with higher power distance, the participants highlighted their need for professional behavior guidelines—a code and conduct that could be used by students and faculty members to standardize the teaching of professional behaviors. The development of these professionalism guidelines and policies would serve as a form of institutional support for students in learning humanism and professionalism, in addition to rewards for teachers, policies to promote a professional environment, and a faculty development program for all faculty members [12, 32].

According to the results of this study, the high-power distance and collectivistic cultural dimension influence the process of teaching humanism and professionalism. These cultural dimensions shape interactions between students and teachers. Because students look up to teachers as role models, the generation gap sometimes creates challenges in their daily relationships. This finding resonates with the results of several other studies, especially those conducted in the Eastern context [8, 23, 33, 34].

Cohen and Sherif [35] described 12 tips for teaching humanism, most of which are applied during physician–patient interaction observations to encourage medical students to develop the habit of applying humanism in their daily lives. Another study [7] emphasized small-group teaching, clinical rounds, conversations, and service-learning experiences as learning methods for embracing humanism in medical education. However, the results of this study highlight the generation gaps and the compromising culture of students, which must be acknowledged as potential challenges; these can be mitigated by providing more opportunities for students to engage in teacher-driven conversations with educators to discuss their experiences and reflections [36].

The present study also highlighted that professionalism is best learned through direct patient contact, which allows students to practice compassion, empathy, and respect during clinical encounters. The COVID-19 pandemic tremendously affected teaching and learning in the clinical setting because it limited direct patient contact, which resulted in fewer opportunities for students to receive the required training for humanism development [23]. To overcome this challenge, training students to use telemedicine has become necessary. In particular, encouraging medical students to practice communication in a humanistic way through virtual encounters in daily practice would also support them as they learn the proper way of taking a patient’s medical history. Learning objectives in telemedicine should emphasize empathic communication techniques, such as comforting patients and listening to their complaints [37]. Aside from telemedicine, regarding the latest technology development and rapid adaptation following the pandemic, the respondents also highlighted the importance of explicitly teaching and discussing social media for professional use as future medical doctors; this reflects the concept of “e-professionalism,” which elaborates on how to conduct and convey professionalism through digital media [12, 38].

Furthermore, immersive activities in the community with efficient time for debriefing and reflection and opportunities to observe and be observed by the clinical teachers were also clearly limited; therefore, formative assessment and feedback were lacking during the COVID-19 pandemic, which might have affected patient safety and professionalism [39]. The current tools for professional behavior assessment are mostly embedded in summative assessment and are implicitly used as another accessory for evaluation. Misch [40] proposed a “connoisseur” approach to assessing humanism by accurately recording and compiling observations of behaviors with their complexities and attributes. The use of a longitudinal portfolio, which accommodates the multifaceted and longitudinal nature of professionalism instead of only assessing it at the end of the clinical cycle or course, could be suggested as a similar approach [41, 42]. Multiple assessments embedded in other assessment and learning activities, along with hurdle assessment approaches, should also be used to assess professionalism (i.e., workplace-based assessment, the OSCE, simulation, reviews of patient complaints and feedback, knowledge tests, and reports of critical incidents) and prevent fake professionalism [43, 44]. The results of the current study also highlighted the teacher’s role as a coach or mentor in assessing and guiding the development of students’ humanistic and professional behaviors.

In developing teaching-learning framework for nurturing humanism and professionalism, there are several things to be considered: (1) the need for an explicit and longitudinal curriculum on professionalism incorporated into the stages of PIF, the role of a humanistic learning environment, and the faculty development of teachers to accommodate this need; (2) the high power distance culture emphasizes the importance of a teacher-driven approach in initiating changes in the curriculum and learning environment [23, 26], thus highlighting the significance of faculty development; (3) clinical teachers must also emphasize constructive feedback, provide remediation as needed, and ensure continued practice to nurture the humanism and professionalism of clinical students [35].

One limitation of this study is its multicenter qualitative design that involves public institutions with adequate accreditation and established teaching hospitals, as clinical learning takes place not only in teaching hospitals but also in remote teaching hospitals and private institutions. However, by involving and collaborating with three different institutions across the main islands in Indonesia, this study provides an accurate depiction of the dynamic process of nurturing humanism and professionalism in the clinical phase of undergraduate medical education from the perspectives of students, teachers, and program coordinators in a high power distance and collectivist cultural context. As this study focused on undergraduate students in clinical rotation, further studies could explore on nurturing humanism and professionalism in other clinical settings, such as postgraduate residency training.

## Conclusion

Nurturing humanism and professionalism in undergraduate clinical settings in collectivistic and high power distance cultural settings is considered an attempt to build students’ character. This process requires the acknowledgment of students’ PIF as the center of learning humanism and professionalism. The process of learning humanism and professionalism should be conducted longitudinally, explicitly, and dynamically within the curriculum, complemented by patient involvement and the inclusion of newly emerging domains of professionalism, such as technology and social media. Moreover, longitudinal assessments must also be conducted to further induce professional behavior. This framework should be considered in teaching humanism and professionalism and conducting faculty development efforts to nurture humanistic and professional physicians.

## Supporting information

S1 File Emerged themes and subthemes. (DOCX)
