# Supplementary material for: Nurturing humanism and professionalism in a clinical setting: A multicenter study to develop a framework for a learning module for clinical students
Source: PLoS One. 2024 Nov 22;19(11):e0313525. doi: 10.1371/journal.pone.0313525 (PMC11584111; doi:10.1371/journal.pone.0313525)
Supplement: S1 File — (DOCX) [file pone.0313525.s001.docx]

**Emerged Themes and Subthemes**

| **Themes** | **Subthemes** | **Quote** |
| --- | --- | --- |
| Professional identity formation as the center of learning humanism and professionalism | Students’ backgrounds and personal beliefs in the PIF process | *“Students also have their own opinions and standards, including beliefs and religions. Now that could pose a barrier in interacting with other people. For example, when I interact with patients of the opposite sex, I feel reluctant, especially when I have to examine the patient. Maybe it’s something worth considering that some students might feel that way.” (MS, FGD7)* |
|  | Students’ personal aspects (i.e. motivation, resilience, well-being, ability to adapt to change) in the PIF process | *“I think the students are still…far from being a professional…because they still have to adapt [to the clinical phase]. They used to only listen to lectures, and now they have to go to the hospital; it takes time to adjust themselves. Usually, in their first year [of clinical rotations], they’re a bit puzzled and nervous when meeting patients and clinical teachers.” (CT, FGD9)* |
|  | Socialization process as the center of PIF | *“Professionalism is not taught with words, but in the form of following examples [role models], in interacting with patients, interacting and working together with other health professions to treat the patient. So, we learn by seeing and following them.” (MS, FGD8)* |
|  | Attributes and key features of humanistic and professional doctors as the end goal of PIF: |  |
|  | 1. Adequate clinical skills | *“Professional doctors are able to apply their knowledge in medicine comprehensively to the patient.” (PC, FGD11)* |
|  | 1. Humanistic qualities | *“Because we are treating patients, we need to have humanistic values. We need to treat and value patients as humans. Therefore, we need to put ourselves in their shoes and treat them just like how we want ourselves to be treated.” (MS, FGD7)* |
|  | 1. Maintaining competence and lifelong learning | *“A professional doctor also means that they are aware of what they are lacking and are willing to improve themselves in the future. What matters is the mindset that we can still improve ourselves, and there are a lot of things we need to learn.” (MS, FGD3)* |
| Principles of Humanism and Professionalism for the Clinical Clerkship Curricula | Nurturing humanism and professionalism as a character-building process | *“A very important note, what students really need in nurturing professionalism is character. Building students' characters is the main point.” (CT, FGD4)* |
|  | Societal demands of professional doctors as the background of learning process | *“The public demands what kind of qualities [possessed] by doctors in Indonesia that are deemed professional. I think that becomes a factor that motivates us to maintain professionalism. At least we’d know what kind of doctor the public wants. So, we have to improve ourselves [in terms of professionalism] and improve our education on that aspect.” (CT, FGD6)* |
|  | 1. Acting professionally in the era of social media and rapid development of technology | *“Technology is growing fast and there are lots of medical technologies, [smartphone] applications, telemedicine—maybe one of the qualities that current doctors need is humanistic values. Therefore, what makes us different as doctors, whether through face-to-face or through telemedicine applications, are those humanistic values.” (MS, FGD7)* |
|  | Nurturing humanism and professionalism as a longitudinal process | *“I agree that it [teaching professionalism] should be conducted longitudinally. It’ll be more effective if we can insert it into each rotation, like internal medicine and child health, so that it [professionalism] will be more specific and applicable.” (MS, FGD2)* |
| Impact of the cultural domain of learning environments in clinical settings | Unavailability of professional behavior guidelines for stakeholders in the learning environment | *“It [professionalism] is not easy. Because we’re assessing attitudes and behaviors, it’s not like we’re assessing written exams. Maybe we need some sort of guideline for which kinds of behavior are still acceptable and can be corrected, and which kinds of behavior cannot be accepted and are a sign that this student can’t become a medical doctor.” (CT, FGD6)* |
|  | Culture of compromising unprofessional behaviors | *“There’s a tendency to make unprofessional behavior somewhat acceptable… a lot of people [in the clinical environment] think it’s okay, and it makes us [students] think that kind of [unprofessional] behavior is okay too, even though in the beginning we know it’s unacceptable.” (MS, FGD2)* |
|  | Generation gaps and hierarchical culture in clinical settings | *“From what I see… they [students] are pressured. Clinical teachers pressured residents; residents pressured medical students. It’s like consecutive pressures.” (CT, FGD14)* |
|  | Limited opportunities for interprofessional collaborative practice in the learning environment | *“We meet a lot of people in the hospital, not only doctors, but also nursing, midwifery, or physiotherapy students. But, in reality, we don’t have any sessions or activities that involve interacting with them. Real nurses, on the other hand, are, in a way, our teachers too. We can’t really collaborate with them, because they’re our teachers.” (MS, FGD8)* |
|  | Nurturing humanism and professionalism as “the second-layer teaching agenda” | *“Yes, we are more focused on the clinical aspect of the case. About the professionalism aspect...sometimes we skipped it a little, sorry.” (CT, FGD10)* |
| Teaching and Learning Humanism and Professionalism Strategies | Meaningful integrated learning experiences with direct patient interaction | *“Professionalism can be taught [and] integrated with the ongoing clinical rotations, where students can interact with patients, participate in bedside teachings, and be assessed with miniCEX.” (CT, FGD4)* |
|  | Immersive intra- or extracurricular community activities | *“In the community health rotation, I feel more professional to patients, to the community. We were taught to work with the community, give education to the elderly, etc. We focused on preventive measures, so even though we didn’t learn much clinical knowledge, we felt connected to the community and learned how to become good doctors among the community.” (MS, FGD8)* |
|  | Reflection and debriefing sessions of learning experiences to internalize humanism and professionalism | *“It’d be nice if we had dedicated sessions to talk about students’ experiences in the clinical learning environment. But the session has to be safe, so it can be a safe space for them, and it’s guaranteed that what is said in that room stays in there, so they can freely talk about negative experiences. The goal is to give feedback and affirmations about role models’ behaviors, positive and negative ones.” (CT, FGD6)* |
| Longitudinal Assessments to Induce Professional Behaviors in Clinical Settings | Professionalism as an embedded component in other assessments |  |
|  | 1. Implicit nature of assessment of professionalism | *“From what I know, professionalism is one of the aspects that is being assessed in clinical examinations, but I feel that in these two years [of clinical clerkship], the subject of professionalism is not really touched upon…and not consistent.” (MS, FGD3)* |
|  | 1. The issue of fake professionalism | *“We all know about fake professionalism. In front of patients, they are doing and saying things that they don’t really mean with sincerity.” (CT, FGD6)* |
|  | 1. Professionalism is not prioritized in assessments | *“A lot of students think that they only need to pass the national competency exam. However, humanism and professionalism are not [explicitly] stated in the national competence exam…our students are very pragmatic.” (PC, FGD11)* |
|  | Longitudinal assessment approach |  |
|  | 1. The importance of hurdle assessment | *“[Professionalism on national competency exam] When it’s not there…our students still need the external ‘pressure’ to increase their motivation to learn something. When it [professionalism] is not a requirement, it’s going to be hard.” (PC, FGD11)* |
|  | 1. Applying portfolios as learning and assessment instrument | *“I don’t know if we can see [students’ progress] from year one to year two if it’s getting better from the start to the end of the clinical rotation. Hopefully, clinical rotation can give color and strengthen students’ professionalism. From the beginning of clinical clerkship until the end, when they’re preparing for the national competency exam.” (PC, FGD5)* |
